# A Newly Developed Sublingual Tonometric Method for the Evaluation of Tissue Perfusion and Its Validation *In Vitro* and in Healthy Persons *In Vivo* and the Results of the Measurements in COPD Patients

**DOI:** 10.1155/2014/534130

**Published:** 2014-12-16

**Authors:** Zoltán Rózsavölgyi, Domokos Boda, Andrea Hajnal, Krisztina Boda, Attila Somfay

**Affiliations:** ^1^Department of Pulmonology, University of Szeged, 36 Alkotmány Street, Deszk 6772, Hungary; ^2^Department of Pediatrics, University of Szeged, Korányi Fasor 14, Szeged 6720, Hungary; ^3^Department of Medical Physics and Informatics, University of Szeged, 9 Korányi Fasor, Szeged 6720, Hungary

## Abstract

*Introduction*. Since its first publication in the medical literature, an extremely large number of references have demonstrated that the tonometric measurement of tissue perfusion is a reliable indicator of the actual condition of critically ill patients. Later a new method was developed by the introduction of sublingual tonometry for the determination of tissue perfusion. In comparison with gastric tonometry, the new method was simpler and could even be used in awake patients. Unfortunately, at present, because of severe failures of manufacturing, the device is withdrawn from commerce. *Materials and Methods*. In this study, we present a new method using a newly developed tool for the P_sl_CO_2_ measurement in sublingual tonometry as well as the data for its validation *in vitro* and *in vivo* and the results of 25 volunteers and 54 COPD patients belonging to different GOLD groups at their hospitalization due to the acute exacerbation of the disease but already in a stable condition at the time of the examination. *Results and Conclusion*. The results of the performed examinations showed that the method is suitable for monitoring the actual condition of the patients by mucosal perfusion tonometry in the sublingual region.

## 1. Introduction

Since its first publication in 1959 [1], an extremely large number of references have demonstrated that the tonometric measurement of tissue perfusion is a reliable indicator of the actual condition of critically ill patients [2–4] and can even be used to predict their morbidity and mortality. The principle of the method is that the tissue PCO_2_ (P_t_CO_2_) of the circulating blood measured inside the mucosa of the actual organ rises sharply with the failure of tissue perfusion. In general this was performed using a ballooned gastric catheter [5–8].

A new method was developed by the introduction of sublingual tonometry for the determination of tissue perfusion [9–11]. In comparison with gastric tonometry, the new method was simpler and could even be used in fully conscious patients [12–18]. Previous studies demonstrated high correlation between sublingual tissue PCO_2_  (P_sl_CO_2_) and gastric tissue PCO_2_ (P_g_CO_2_) in various conditions, confirming sublingual tonometry as a rapid, minimally invasive method.

Unfortunately, in spite of these advantages, at present, owing to severe failures of manufacturing, the device serving for the sublingual tonometry has been withdrawn from the commerce.

In this study, we present a new method using a newly developed device for the measurement of the P_sl_CO_2_ in sublingual tonometry as well as the data for its validation and the first results in COPD patients in a stable condition following recovery after the exacerbation of the disease.

## 2. Materials and Methods

The study was registered on http://www.clinicaltrials.gov/ (ID: NCT01169506). The examinations were approved by the Human Investigation Review Board of the University of Szeged, Hungary (number 2497. 23.03.2009). All the volunteers and patients were fully informed and their written consent was obtained.

### 2.1. Materials

**Figure 1:**
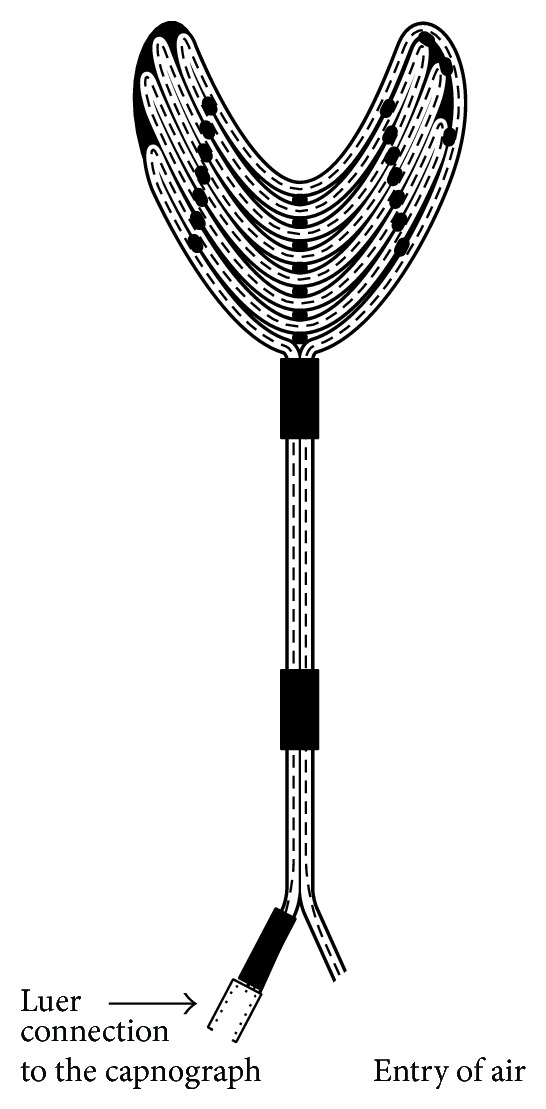
Scheme of the sublingual tonometer.

*The New Sublingual Probe*. The basic tool for P_sl_CO_2_ measurement is a probe: a specially coiled silicone rubber tube produced by Medisintech Ltd., Budapest, Hungary. The gas content (room air) inside the tube of the sublingually inserted probe gets in equilibrium by diffusion with the gases of the blood circulating in the capillaries of the sublingual mucosa. Then it is transferred into a capnograph (Oridion Microcap, Oridion Capnography Inc., Needham, MA, USA) to measure its CO_2_ content. During the production process the silicon rubber is moulded into a butterfly shape. In order to prevent the obstruction of the lumen by kinking, a 0.3 mm thick polyamide fibre is inserted along the tube; thereby after folding the tube, sufficient lumen volume remains to allow the filling test material to be transported into the measuring unit (Figure 1).

### 2.2. Methods

#### 2.2.1. Technique of the Measurement of the P_sl_CO_2_

The probe is kept under the patient's tongue between the tongue and the sublingual mucosa in such a way that it reaches the end of the frenulum linguae. During the measurement the patient is not allowed to breathe through the mouth until the full equilibration of the sublingual probe (15 minutes). Then the probe is connected to a capnometer via a Luer lock connection, and its gas content is transferred into it.

#### 2.2.2. *In Vitro* Validation Method

To measure the* in vitro *CO_2_ uptake into the probe, a glass container was used for equilibration. The probe was inserted into this container up to the Luer connector part of the device and the container was closed in an airtight manner. Then it was perfused with air containing CO_2_ at 35 mmHg, provided from gas cylinders at a flow rate of 5 L/min. The* in vitro* uptake of CO_2_ of the probe was tested at room temperature and at 37°C as well, when the equilibration chamber was submerged in thermostated water of constant temperature. Room air was used as filling media. At each control time of the CO_2_ uptake of the probe, four parallel measurements were made. The PCO_2_ value of the container was checked every 10 minutes. These values were used as references for the* in vitro* measurement data. After the defined time intervals, the filling medium was aspirated and displayed by the capnograph. All* in vitro *measurement data obtained after the given equilibration times were expressed as percentages of the reference value.

#### 2.2.3. Simultaneous Determination of P_a_CO_2_ (P_c_CO_2_)

Realizing the difficulties in sampling of arterial blood we used arterialized capillary blood samples for the measurement in the study [19]. For this purpose blood was taken from the earlobe of each individual following local application of Finalgon (Boehringer Ingelheim GmbH, Ingelheim am Rhein, Germany) for 15 minutes. The blood samples were analysed by Radiometer ABL5, Radiometer Medical ApS, Brønshøj, Denmark.

#### 2.2.4. *In Vivo* Validation Method

The* in vivo* CO_2_ uptake by the probe was measured performing 25 sublingual tonometries in five healthy volunteers. Each volunteer underwent five measurements which lasted 1, 2, 6, 10, and 15 minutes, respectively. Each measurement was followed by a two-minute pause. In these experiments the highest measured value obtained at 15 minutes' equilibration time served as reference value and to express the formerly measured data in percentages.

#### 2.2.5. Controlling Rapid Changes during Hyperventilation of the Sublingual Tonometric Values

P_sl_CO_2_, P_c_CO_2_ values were determined in healthy volunteers while ventilating normally for 5 minutes and during 5 minutes of hyperventilation.

#### 2.2.6. Parallel Sublingual Tonometric Measurements

In order to test the consistency of the method, in 42 out of the 54 patients, parallel P_sl_CO_2_ measurements were also carried out. That is, 15 minutes after the end of the first sublingual measurement (regeneration time) the patients placed the probe under their tongue for another 15 minutes' time and the values were determined by the capnometer.

#### 2.2.7. Study Population

25 healthy volunteers were involved in the study. The COPD study population was composed of 54 patients hospitalized with symptoms of acute exacerbation. They were involved in the study only after 7–14 days, following stabilisation of their condition with corticosteroid and/or antibiotic treatment. None of them needed either intubation or supplementary oxygen.

COPD diagnosis was based on medical history and spirometry (postbronchodilator FEV1/FVC < 0.7) on the grounds of GOLD criteria [20]. The following parameters were registered in all patients: age, sex, postbronchodilator FEV_1_, GOLD stages, smoking history, sublingual PCO_2_ (P_sl_CO_2_), value of arterialized capillary blood gas analysis (P_c_O_2_, P_c_CO_2_, pH), and body mass index [21].

#### 2.2.8. Statistical Analysis

All numerical values are expressed as the mean ± SD. One-way analysis of variance (ANOVA) and post hoc analysis (LSD test with Sidak adjustment for multiple comparisons) were used for the comparison of the four groups, that is, the control group, COPD II, COPD III, and COPD IV. Paired* t*-test was used to evaluate differences in control P_sl_CO_2_ versus P_c_CO_2_. The relationship between parallel measurements was examined by Pearson's correlation. Normal ventilation to 5 min and hyperventilation to 5 min were also compared by paired* t*-test. A value of *P* < 0.05 was considered to be statistically significant. SPSS 15.0 was used for statistical calculations.

## 3. Results

### 3.1. Clinical Data of the Study Population at Hospitalization

Table 1 shows the clinical data of the COPD patients at the time of their admission.

### 3.2. Experimental* In Vitro* and* In Vivo* Measurements

**Figure 2:**
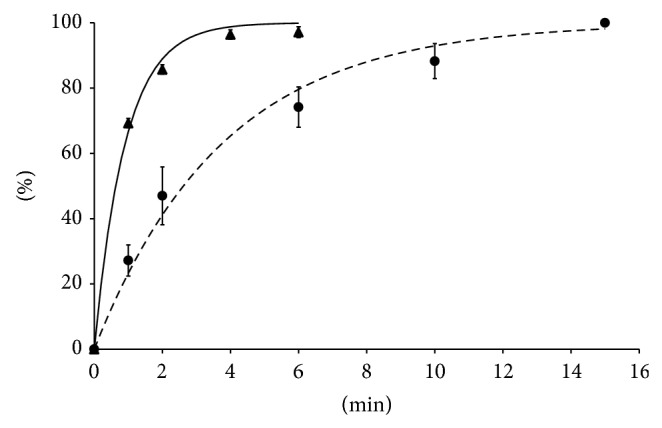
Experimental CO_2_ uptake by the sublingual probe after given equilibration times. Values expressed as percentages of the reference data.* In vitro* at 37°C (▲), equation: *y* = 100(1 − *e* ^−1.101*x*^), and* in vivo* (●), equation: *y* = 100(1 − *e* ^−0.265*x*^).

The data of experimental* in vitro* CO_2_ uptake by the probe at 37°C after given equilibration times are expressed as percentages of the reference data and are presented in Figure 2. Nearly the same results were obtained by performing the measurement at room temperature (data not shown). These data show that the time required for the* in vitro* CO_2_ uptake from the closed equilibration chamber into the probe is very short. Equilibrium is virtually complete within 4 minutes in room air, as filling medium. However, the time required for equilibrium to be attained in* in vivo *measurements was significantly longer. Full equilibrium with room air within the probe was achieved in 15 minutes.

### 3.3. P_sl_CO_2_, P_c_CO_2_ Measurements in Healthy Control and COPD Patients

The mean values of P_sl_CO_2_ and P_c_CO_2_ did not differ significantly either in healthy individuals (39.7 ± 2.8 versus 37.7 ± 1.3 mmHg) or in patients with COPD (44.4 ± 9.3 versus 42.7 ± 7.7 mmHg), respectively. The difference between P_sl_CO_2_ and P_c_CO_2_ did not change significantly in different severity stages (GOLD stages I–IV) of COPD. Moreover, the mean values increased as the stage of COPD was more severe (test for linearity, *P* < 0.0001). Normal pH indicated that the patients were in a stable phase of the disease (Table 2).

### 3.4. Effect of Hyperventilation in Healthy Volunteers

Similarly to previous studies [17], hyperventilation resulted in significant decrease in values also using our method. The mean difference between P_sl_CO_2_ measured after 5 minutes' equilibration at normal ventilation and after 5 minutes of voluntary hyperventilation was statistically significant (mean difference 6.4 ± 6.8 mmHg, 95% CI 4.49–8.31, *P* < 0.0001) (Table 3).

### 3.5. Results of the Parallel Sublingual Tonometric Measurements

The mean difference between the two tonometric parallel measurements of the P_sl_CO_2_ was 0.81 ± 2.61 mmHg, with a correlation of *r* = 0.958, *P* < 0.0001.

### 3.6. Results of the Parallel Measurements of the Arterial and Arterialized Capillary Blood Gases

Data collected in Table 4 show that the difference between the two studies performed by different methods is not significant in terms of pH and PCO_2_.

## 4. Discussion

The main object of the present study was to work out a method for the determination of sublingual mucosa PCO_2_ (P_sl_CO_2_). According to the results described above we can declare that our technique is suitable for sublingual tissue perfusion measurement. Our data show that the generally accepted characteristic parameter of tissue perfusion, that is, the difference between sublingual mucosa PCO_2_ and blood PCO_2_ known as gap value measured using the new device, basically corresponds to the earlier results of conventional studies. P_sl_CO_2_ values were higher only in patients who were diagnosed with stage IV COPD at the admission to the department. However, at the time of the examination, a few weeks later, following recovery, they were also in a stable condition and the higher P_sl_CO_2_ value as compared to the other groups and the compensated respiratory data were the only indicators of the original stage IV COPD but the gaps of the measured values in this patient population group were not significantly more elevated than those measured in patients with II and III GOLD stages.

The observation that P_sl_CO_2_ values were considerably lower during spontaneous hyperventilation shows that the method is suitable for the follow-up of relatively short term changes in the P_(a)sl_CO_2_ values. Concordance of the corresponding data of parallel measurements also supports the reliability of our method.

On the other hand, the fact that the test takes 15 minutes can be inconvenient for the patients and it can contraindicate the examination during acute exacerbation of the disease, which limits considerably the field of application of the present method.

The use of arterialized capillary blood samples instead of arterial blood can also be considered as a drawback of our method. The reliability of substitution of arterial blood samples with arterialized capillary blood is supported by a large body of scientific evidence in the literature [19] although in some studies negative opinions can also be met. There is no doubt that in this type of examination the use of arterial blood must be kept as the gold standard. We had to put this rule aside because of the need for a highly cautious method required for the determination of P_sl_CO_2_. In order to counterbalance the disadvantages we applied the method of the highest standard for the examination of the arterialized capillary blood. Moreover, we compared the values of the different groups of our patients. The data in Table 4 show that the difference in terms of pH and PCO_2_ is not significant.

## 5. Conclusion

Based on the results of the study, we conclude that our method is a reliable new mean of sublingual tissue PCO_2_ determination. The method cannot be recommended for everyday clinical practice as, for example, in shock or other acute life-threatening conditions, because of the time-consuming nature of the examination. Nevertheless, the technique can be a useful tool in the differential diagnosis of some diseases, such as panic disorder and pseudoasthma, or in the determination of CO_2_ accumulation in patients on continuous oxygen therapy. We also think that our results may provide basis for further development and investigations in this field.

To give judgement on the validity of the modified technique further extended examinations are required.

## Figures and Tables

**Table 1:** Clinical data of the study population.

COPD GOLD groups	II	III	IV
Number	15	22	17
Sex (male/female) number	9/6	13/9	14/3
Age, years	60.7 ± 8.2	63 ± 16.2	57 ± 8.1
Body weight	77.6 ± 24.0	65.8 ± 16.2	77.2 ± 16.5
Body mass index (kg/m^2^)	26.4 ± 7.2	23.4 ± 4.0	27.1 ± 5.8
O_2_ therapy (+/−)	3/12	5/17	15/2
Smoking, pack-year	41.8 ± 24.1	36.7 ± 26.3	38.3 ± 23.5

Values given as mean ± SD.

**Table 2:** Sublingual PCO_2_, arterialized capillary blood PCO_2_, and pH values in healthy controls and COPD patients.

	P_sl_CO_2_ mmHg^*^	P_c_CO_2_ mmHg^*^	P_sl_CO_2_ − P_c_CO_2_ mmHg^#^	pH^#^
Healthy controls				
Number	10	10	10	10
Mean ± SD	39.7 ± 2.8	37.7 ± 1.3^e^	2.0 ± 3.1	7.43 ± 0.02
COPD stage II				
Number	15	15	15	15
Mean ± SD	37.4 ± 5.2	36.6 ± 3.0	0.8 ± 4.5	7.43 ± 0.02
COPD stage III				
Number	22	22	22	22
Mean ± SD	43.5 ± 8.1	41.2 ± 4.6	2.3 ± 6.7	7.43 ± 0.02
COPD stage IV				
Number	17	17	17	17
Mean ± SD	51.6 ± 8.8^a^	49.9 ± 8.2^a^	1.6 ± 4.2	7.41 ± 0.03
COPD in all cases				
Number	54	54	54	54
Mean ± SD	44.4 ± 9.3	42.7 ± 7.7	1.7 ± 5.4	7.42 ± 0.02

P_sl_CO_2_, carbon dioxide sublingual partial pressure; P_c_CO_2_, carbon dioxide arterialized capillary partial pressure; _c_pH, arterialized capillary pH;  ^*^ *P* < 0.0001, ANOVA result for comparison of the four groups;  ^#^ *P* > 0.05, ANOVA result for comparison of the four groups;  ^a^ *P* < 0.001, compared to the other groups; ^b^ *P* < 0.05, compared to COPD II and COPD III; ^c^ *P* < 0.0001, compared to COPD II and COPD III; ^d^ *P* < 0.001, compared to COPD III and COPD IV; ^e^ *P* > 0.05, compared to P_sl_CO_2_.

**Table 3:** Testing under hyperventilation in healthy volunteers.

	P_sl_CO_2_ mmHg	P_c_CO_2_ mmHg
Control, normal ventilation to 5 min.		
Number	10	10
Mean ± SD	27.2 ± 3.1	37.7 ± 1.3
Control, hyperventilation to 5 min.		
Number	10	10
Mean ± SD	20.8 ± 3.4	20.4 ± 2.8

P_sl_CO_2_, carbon dioxide sublingual partial pressure; P_c_CO_2_, carbon dioxide arterialized capillary partial pressure.

**Table 4:** Differences of blood gas parameters measured in the simultaneously sampled arterial  blood and of the arterialized capillary blood in COPD patient with various conditions.

Differences in	pH	PCO_2_ mmHg
Number	15	15
Mean ± SD	0.0013 ± 0.011	0.333 ± 1.345
Max–min	0.02–0	3–0

## Abbreviations

ANOVA: Analysis of variance
CI: Confidence interval
CO_2_: Carbon dioxide
COPD: Chronic obstructive lung disease
FEV1: Forced expiratory volume/sec
FVC: Forced vital capacity
GOLD: Global Initiative for Chronic Obstructive Lung Disease
ID: Internal diameter
LSD: Least significant difference
OD: Outside diameter
PCO_2_: Carbon dioxide partial pressure
${\text{P}}_{\text{a}}{\text{CO}}_{2}$: Carbon dioxide arterial partial pressure
${\text{P}}_{\text{c}}{\text{CO}}_{2}$: Carbon dioxide arterialized capillary partial pressure
${\text{P}}_{\text{g}}{\text{CO}}_{2}$: Carbon dioxide partial pressure of gastric mucosa
${\text{P}}_{\text{sl}}{\text{CO}}_{2}$: Carbon dioxide partial pressure of sublingual mucosa
${\text{P}}_{\text{t}}{\text{CO}}_{2}$: Carbon dioxide partial pressure of tissue mucosa
${\text{P}}_{\text{a}}{\text{O}}_{2}$: Blood oxygen partial pressure
SPSS: Statistical Software for Social Sciences.
